# Representation Learning Method with Semantic Propagation on Text-Augmented Knowledge Graphs

**DOI:** 10.1155/2022/1438047

**Published:** 2022-09-27

**Authors:** Ling Wang, Jicang Lu, Gang Zhou, Hangyu Pan, Taojie Zhu, Ningbo Huang, Peng He

**Affiliations:** State Key Laboratory of Mathematical Engineering and Advanced Computing, Information Engineering University, Zhengzhou 450001, China

## Abstract

Knowledge graph representation learning aims to provide accurate entity and relation representations for tasks such as intelligent question answering and recommendation systems. Existing representation learning methods, which only consider triples, are not sufficiently accurate, so some methods use external auxiliary information such as text, type, and time to improve performance. However, they often encode this information independently, which makes it challenging to fully integrate this information with the knowledge graph at a semantic level. In this study, we propose a method called SP-TAG, which realizes the semantic propagation on text-augmented knowledge graphs. Specifically, SP-TAG constructs a text-augmented knowledge graph by extracting named entities from text descriptions and connecting them with the corresponding entities. Then, SP-TAG uses a graph convolutional network to propagate semantic information between the entities and new named entities so that the text and triple structure are fully integrated. The results of experiments on multiple benchmark datasets show that SP-TAG attains competitive performance. When the number of training samples is limited, SP-TAG maintains its high performance, verifying the importance of text augmentation and semantic propagation.

## 1. Introduction

Knowledge graphs (KGs) are structured graph databases, usually large in scale, with many entities, relations, and triples. KGs are useful for intelligent search [1], recommendation systems [2, 3], intelligent question answering [4, 5], and other applications. Common KGs include Freebase [6], YAGO [7], and WordNet [8]. Although these KGs are huge in scale and cover a wide range of information, they still face problems such as missing data and incomplete semantics, limiting the performance of subsequent applications. Therefore, completing the missing facts semantically in KGs is an important task.

KG representation learning (KGRL) is an effective and practical approach to predicting missing facts. In recent years, it becomes an important research direction in KG. KGRL embeds entities and relations in KG into vectors and then predicts potential triples through vector computation. In early KGRL methods such as TransE [9] and TransH [10], researchers embedded entities and relations using only triples; however, the representation ability of these methods for complex relations is limited. Hence, researchers have proposed various improvements, such as RotatE [11] and ConvE [12]. However, these methods may have difficulty in training when the number of triples with specific entities or relations in KG is limited. The model may not fully learn the features of KG elements through limited training samples, thus affecting training effect of the model.

To solve this problem, many methods integrate external auxiliary information such as type, time, text, or images into the model. They usually encode auxiliary information or expand triples into “quadruples,” endowing the entity with rich information to compensate for the lack of training samples. Text corpora contain more semantic information. Researchers have proposed a variety of methods for text, such as NTN [13], DKRL [14], ConMask [15], and TEGER [16]. These methods integrate semantic information within text into entity representations to improve the representation performance but retain the following shortcomings:

1. Text information does bring semantic supplement to entities. Existing methods tend to encode the text information independent of the triple structure, which does not combine semantic and triple structure information well. Moreover, the sparsity of KGs, which make it difficult to share semantic information between related entities, remains a problem.
2. Entities in the same triple tend to be semantically associated. Modelling the association information can obtain the features at entity-pair level and be beneficial to representation learning. However, existing methods inadequately represent such associations.
3. These methods generally use traditional word embeddings to encode text information. For one token, there will be only one representation even in different contexts. The lack of semantic information is insufficient for encoding text description sequences.

In this study, we propose SP-TAG, a KGRL method with semantic propagation on text-augmented knowledge graph. SP-TAG embeds the triple structure and text description information in the same semantic space. Named entity recognition (NER) [17], as well as relation classification (RC) [18, 19], is an important task in process of constructing KGs from unstructured text. Inspired by the process, for each entity in KG, we first extract the entities from the text description (called text entities, corresponding to the existing entities in the knowledge graph, i.e., original entities) with NER. The text entities are then connected to the original entities in the KG to construct a text-augmented KG, which contains the information from both triples and text. We then use a pretrained language model to obtain the initial feature embedding of the nodes in the text-augmented KG, which incorporates semantic information into the model. Next, a graph convolutional network (GCN) [20] is used to propagate the semantic features of the entities so that the text-based representation contains semantic information from other nodes. Finally, we jointly learn the structure- and text-based representations of the entities using a gate mechanism in the same vector space.

The main contributions of this study are as follows:

1. The proposed SP-TAG extracts text entity nodes to construct a text-augmented KG based on NER from the text description of the entity, which increases the average number of edges around the entity and reduces sparsity.
2. SP-TAG uses a pretrained language model to initialize the semantic features of the nodes. It also uses a GCN to propagate semantic features among entities to better integrate structural and textual information, thereby improving the semantic associations between entities. SP-TAG can be combined with existing KGRL methods to improve performance.
3. We conducted experiments on multiple benchmark datasets to compare SP-TAG with methods that only consider triples or integrate text information. The results also show that SP-TAG is more reliable with few training samples because of its augmentation and propagation characteristics.

## 2. Related Works

This section reviews three types of KGRL methods: methods that only use triples, methods that integrate text information, and new key techniques we use in our method, i.e., GCNs.

### 2.1. Methods Based on Triples

**Figure 1:**
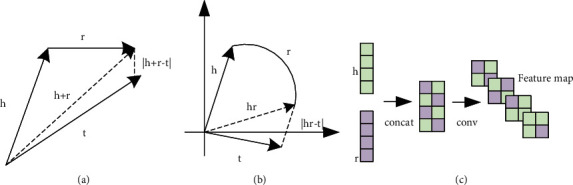
KGRL methods based on (a) translation, (b) rotation, and (c) neural networks.

These methods can be divided into methods based on translation, rotation, and neural networks (Figure 1). A classic translation method is TransE [9]. For each triple, TransE considers the relation *r* to be a translation operation from head entity *h* to tail entity *t* in vector space and uses a distance function to calculate the score, which in turn measures the confidence that the triple is true. The score function is as follows:

(1) $$\begin{array}{c} f_{r}\left(h,t\right)=-{\;\left\|h+r-t\right\|}_{1/2}\text{.} \end{array}$$

TransE cannot effectively deal with complex relations such as 1-to-*N*, *N*-to-1, and *N*-to-*N* relations. TransH [10], TransR [21], and TransD [22] employ different projection strategies to improve the representation ability of this approach and handle such complex relations. TransH [10] maps the head and tail entity to different relation hyperplanes for further calculation; TransR [21] embeds entities and relations into entity and relation spaces, respectively, and TransD [22] constructs a dynamic mapping matrix to reduce the number of parameters of TransR.

The concept in rotation methods originates from Euler's formula, *e*^*iθ*^=cos *θ*+*i* sin *θ*, which is used to embed entities and relations into complex space. In RotatE [11], for each triple, *t* is obtained from *h* through a rotation operation of relation *r*, which greatly improves its ability to represent symmetry relations such as “classmates.” This method also measures the confidence of the triple by calculating the distance, as follows:

(2) $$\begin{array}{c} f_{r}\left(h,t\right)=-{\;\left\|h\circ r-t\right\|}_{1}\text{.} \end{array}$$

QuatE [23] improves on RotatE by extending the complex space to quaternion space. MRotatE [24] combines both entity and relation rotations to further improve its ability to represent complex relations.

Many KGRL models are based on neural networks. Typical models include models that extract the deep features of triples using convolutional neural networks (CNNs) such as ConvE [12] and ConvKB [25]; models that learn long-distance KG relation dependencies using recurrent neural networks (RNNs) such as RSN [26]; models that generate trajectory sequences by traversing KGs using generative adversarial networks (GANs) such as GRL [27]. These models perform well on real datasets, but the geometric interpretation is not as clear as it is in translation- and rotation-based methods. Because a neural network is a “black box,” [28] its interpretability is not strong.

Triple-based models perform reasonably well, but they often require sufficient training samples and are thus susceptible to sparsity in the KG. Moreover, such models do not fully utilize auxiliary information, making it difficult to accurately represent entity and relation semantics.

### 2.2. Methods Integrating Text Information

In some KGs, entities have related text description information that does not exist in the triples, thus complementing the representation of the entities. To better utilize this information, existing methods usually encode it as a vector (referred to as *text-based representations* here) for joint training with the triple-based entity representations (referred to as *structure-based representations* here). Researchers originally used a one-hot or co-occurrence matrix to encode text, but as the size of the text increases, the computational complexity and number of parameters rapidly increase, and hence this approach is not suitable for large corpora. The word embedding method Word2VEC [29] maps words into a lower-dimensional dense vector that retains text features and greatly reduces the computational complexity. In recent years, with the development of deep learning, CNNs [30], RNNs, and BERT [31] are often used to learn and extract deep semantic features from text. BERT is a pretrained model based on a multilayer transformer architecture. It captures the contextual information of text sequences and represents semantics accurately and efficiently.

Models that integrate text information include DKRL [14] and Joint [32], which use a continuous bag of words and CNN for text encoding; STKRL [33], which uses an RNN to obtain text sequence information; EDGE [34] and AATE [35], which are based on the bidirectional long short-term memory network; TA-ConvKB [36], which uses bidirectional short and long term memory network with attention to encode the text. The text- and structure-based representations are generally combined when calculating the score functions. DKRL fuses the two representations using interleaving operations. Joint and AATE fuse the representations using combination mechanisms such as a gate structure and weight parameters. TA-ConvKB uses LSTM to combine the representations. Pretrain-KGE [37] utilizes BERT to obtain textual representations of entities and relations, introducing semantic information into the triples.

Some models do not adopt the idea of a joint representation. For instance, ConMask [15] allocates attention by calculating the semantic similarity of each word in the head entity description and relation, fuses the representation of the head entity description word and relation representation to extract features, matches tail entities, and ranks entities according to similarity. ConMask does not exploit the structural information of triples. OWE [38] establishes a transformation matrix between the structure and text representation space and converts the text-based representation into a structure-based representation to calculate scores. KG-BERT [39] treats triple reasoning as a sequence prediction problem. By inputting all entity descriptions and relations into the transformer, KG-BERT determines whether the triple is correct according to the result of the sequence output.

These models increase their representation ability by integrating text information, but the text encoding process is often independent of the structure of the KGs. It is difficult to fully integrate the text information and KG structure, and thus the utilization of the semantics in the text remains insufficient. TEGER [16] uses TF-IDF to extract keywords from text descriptions and connects them to the KG to expand the KG. Then, TEGER follows traditional TransE method to obtain the embeddings of entities and relations. It strengthens the connection between text information and knowledge graph and improves the link prediction results.

### 2.3. Graph Convolutional Network (GCN)

A graph is composed of nodes and edges. It is not a structured matrix, and hence traditional CNN methods cannot be used for feature extraction. To extract rich information from graphs, the GCN [20] was proposed. Similar to a CNN, a GCN is essentially an aggregation of operations on the neighborhood information and can be divided into three steps. The nodes send their information to the neighbor nodes, aggregate the information from their neighbors, and perform a nonlinear transform on the aggregated information.

However, a GCN cannot be directly used for KGs because it often ignores edge information, i.e., relations. Relational graph convocational network (R-GCN) [40] introduces a neighbor node aggregation model based on a GCN that considers edge types. SACN [41] divides the entire graph into multiple subgraphs that each contain only one relation and then applies the GCN to each subgraph separately. TransGCN [42] combines the translation model and GCN to learn the representation of entities and relations and integrates the triple scoring function into the model.

Models based on GCNs usually propagate information among the nodes in graph data to obtain more complex node neighbor features, which has strong potential in the field of KGRL.

## 3. Proposed Method

In this study, a KG is denoted as *K*={*E*, *R*, *T*}, where *E*, *R*, and *T* denote the sets of entities, relations, and triples, respectively. Each triple is defined as (*h*, *r*, *t*), where *h*, *r*, and *t* refer to the head entity, relation, and tail entity, respectively. Its vector embeddings are denoted as the bold symbols **h**, **r**, and **t**. The text entity set extracted from the text description is denoted as *e*_*ner*_, and the text-augmented KG is *K*_TAG_. Subscripts *s* and *d* represent the structure-based and text-based representations of the entity, respectively, e.g., e.g., *h*_*s*_, *t*_*s*_, *h*_*d*_, and *t*_*d*_.

As noted in Section 2, most existing methods encode text independently of the triple structural information. This makes these methods only retain the semantic information in the text, while ignoring the connection between the text and the knowledge graph structure. A few methods [16, 32] connect the keywords from the text to the KG, realizing the fusion of text and structure. But on the one hand, these methods do not take into account the heterogeneous features of knowledge graphs during embedding; on the other hand, the methods used to obtain node representations only with triple/graph structure (such as TransE or graph neural networks) without preserving the semantics of the text information.

**Figure 2:**
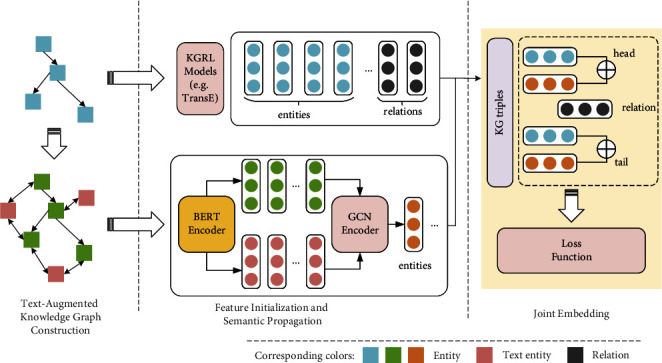
Overall schematic diagram of SP-TAG. This figure is recommended for viewing in color.

In response, we propose a representation learning method with semantic propagation on a text-augmented KG called SP-TAG, combining the text and the structure, and introducing rich semantic information. SP-TAG consists of three parts: text-augmented KG construction, feature initialization and semantic propagation, and joint embedding. SP-TAG is illustrated in Figure 2, in which the upper-left part is the original KG *K*, where the blue squares represent the entities, and the bottom is the corresponding *K*_TAG_, where the green and red squares represent the entities and text entities, respectively. The rounded rectangles with colored circles represent the embedding of the corresponding elements.

### 3.1. Text-Augmented KG Construction

We construct a text-augmented KG by extracting named entities from an entity's text description and connecting them to KG. An entity *e* in a KG usually has a corresponding text description with related information such as associated entities and attributes. We focus on these keywords in the text description and extract them.

Existing TF-IDF methods do not consider the specific content, number, and parts of speech of keywords, leading to potential noise caused by inappropriate keywords. The nodes in the knowledge graph are entities with clear meaning, so we use named entity recognition to get the same meaningful entity nodes (called text entities) from the text. At the same time, considering the heterogeneous character of knowledge graph, we hope that when the extracted text entities are connected to the knowledge graph, different edges can be selected according to the types of the text entities.

We use the open-source natural language processing tool Spacy (https://spacy.io/) to perform NER operations. This is a popular tool that has achieved good results on several evaluation tasks in natural language processing. Spacy can help us complete the preliminary text processing work, allowing us to focus on the research of knowledge graph representation learning. According to the settings of Spacy, we select 11 common entity types and extract these types of text entities from the text description of the original entity. The 11 types are described in Table 1.

Usually, there is an explicit or latent semantic correlation between the text entities and original KG entities. Therefore, we use two-direct edges to connect them to assist two-way semantic propagation. Simultaneously, for new connections, we do not consider the specific semantics of the edges, distinguishing them only by text entity types.

**Figure 3:**
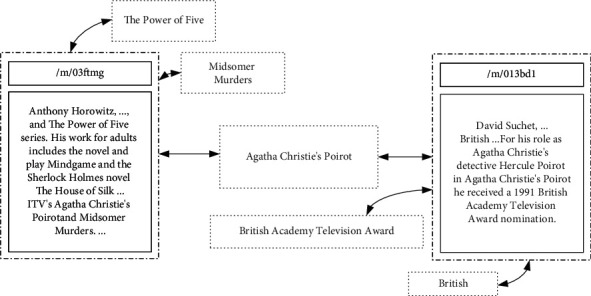
Example of a text-augmented KG. It describes the connection of two entities through a common text entity and implements the process of semantic propagation.

When a text entity is connected to KG, node number around the original entity *e* in KG increases, and the resulting KG is called a text-augmented KG (*K*_TAG_). For example, in Figure 3, “/m/03ftmg” and “/m/013bd1” are the entities in the KG, and the five terms in the dashed rectangles are named (text) entities. Here, two entities not directly connected in the KG acquire a common text entity node neighbor. Therefore, semantic information can be propagated between then using a GCN. The left entity in the figure is the screenwriter “Anthony Horowitz,” whereas the right entity is the actor “David Suchet,” and the middle text entity “Agatha Christie's Poirot” happens to be the name of the TV series associated with them both. In this way, the potential associations of entities in the text can be mined and added to the KG, augmenting their semantic associations.

Note that the number of text entities around each entity may differ, while the number of entities in the original KG connected to a text entity may also differ. For example, “Agatha Christie's Poirot” connects two nodes, whereas all other text entities connect only one node.

In SP-TAG, text entities connected with only one entity are removed from *K*_TAG_. Text entities are extracted to connect two entities in the KG that may have semantic associations but no explicit triple, so that we can propagate semantics between them. If a text entity does not establish a connection between two entities, it does not represent additional necessary information. Retaining it will increase the complexity of the model. Moreover, some text entities, such as country and place names, connect too many entities. The relationships between these entities may not have practical significance, resulting in unnecessary semantics. For example, it is not informative for the entity “parrot” to share semantics with “Silicon Valley” via “United States.” To consider both aspects, we define a threshold *k* in SP-TAG, and only when the number of text entities connected to entities is greater than 1 and less than *k*, are the text entities and their connections preserved. We will carry out experiments on parameter *k*. Details are provided in Section 5.2.

SP-TAG learns the text-based representation of entities from *K*_TAG_. In contrast to existing models that integrate text information, SP-TAG augments the existing KG by extracting named entity nodes. This both alleviates the sparsity of KG and lays a foundation for subsequent joint learning of text- and structure-based representations.

### 3.2. Feature Initialization and Semantic Propagation

SP-TAG adopts the existing classical KGRL methods such as TransE and RotatE to initialize the structure-based representation. This representation, which has more credible structure semantics, is directly derived from the triples in the KG and is key in the prediction and reasoning of missing elements in triples.

SP-TAG initializes text-based representations of entities and text entities in *K*_TAG_. Because each entity has an explicit text description, we use BERT to directly encode the entity description. That is, for the textual description *S*(*e*) of each entity, there is a text-based representation **e**_*d*_=BERT(*S*(*e*)) preserving the semantics of the original description. Since the text entity will learn semantic features along with the entity in the subsequent semantic propagation, to ensure that the representations of the text entity and entity are in the same semantic space, SP-TAG directly inputs the text entity name into BERT to obtain its text-based representation, that is, **e**_*ner*_=BERT(name(*e*_*ner*_)).

After initialization, we continue semantic propagation. During the construction of *K*_TAG_, text entities are only connected to original entities, and no edges will be generated between text entities; therefore, direct semantic propagation only occurs between entities and text entities, and between entities and entities. Hence, we focus on semantic propagation between those entities that do not have explicit triples in *K* but are connected by text entities in *K*_TAG_.

We assume that the correlation between entities in the knowledge graph will decrease as the distance increases. It is meaningless to propagate semantic information from one entity to distant entities. The propagation process itself will bring information attenuation as well. Therefore, it is needed to define a threshold to limit the propagation, as well as reduce the computation and simplify the model. By consulting literature and conducting experiments, we set the threshold as 2.

**Figure 4:**
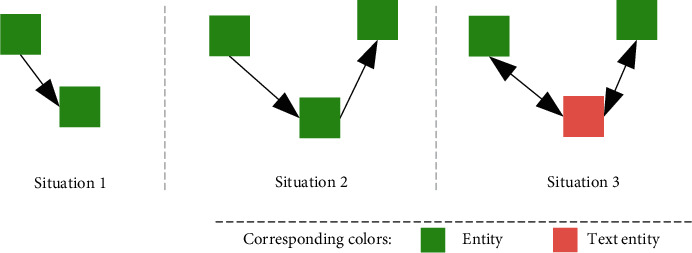
Different situations of semantic propagation in *K*_TAG_. Squares of different colors represent entities and text entities, and arrows represent the process of semantic propagation between these entities.

As shown in Figure 4, there are the following three main situations: (1) entity to entity, (2) entity to entity to entity, and (3) entity to text entity to entity. Situation 1 realizes the close-range semantic propagation between entities with a one-hop connection, situation 2 realizes longer-distance semantic propagation between entities with a two-hop connection, and situation 3 realizes distant semantic propagation between entities connected by a text entity.

Semantic propagation is realized using a simple GCN. The graph convolution operator is expressed as follows:

(3) $$\begin{array}{c} h_{i}^{\left(l+1\right)}=\sigma \left(\underset{j\in N_{i}}{\sum }{\left(1/c_{ij}\right)W^{\left(l\right)}h}_{j}^{\left(l\right)}\right)\text{,} \end{array}$$

where *h*_*i*_^(*l*)^ is the feature vector for node *i* in the *l*th neural network layer, *𝒩*_*i*_ is the set of neighbors of node *i*, *c*_*ij*_ is the normalization constant for edge (*v*_*i*_, *v*_*j*_), *W*^(*l*)^ is a layer-specific weight matrix, and *σ* is the activation function.

Because a KG is essentially a heterogeneous graph, we also considered the use of the more suitable R-GCN for semantic propagation on *K*_TAG_. R-GCN is a variant of the GCN that introduces a specific weight parameter for each relation. Therefore, during semantic propagation, SP-TAG dynamically learns the appropriate weight parameters for different relations to obtain the semantics from neighbor nodes more accurately. We compare the results of GCN and R-GCN in the ablation study.

The overall update process of R-GCN is as follows:

(4) $$\begin{array}{c} h_{i}^{\left(l+1\right)}=\sigma \left(\underset{r\in R}{\sum }\underset{j\in N_{i}^{r}}{\sum }\left(1/c_{ij}^{r}\right)W_{r}^{\left(l\right)}h_{j}^{\left(l\right)}+W_{0}^{\left(l\right)}h_{i}^{\left(l\right)}\right)\text{,} \end{array}$$

where *h*_*i*_^(*l*)^ is the feature vector for node *i* in the *l*th neural network layer, *𝒩*_*i*_^*r*^ is the set of neighbors of node *i* with edge *r*, *c*_*ij*_^*r*^ is the normalization constant for edge (*v*_*i*_, *v*_*j*_) with edge *r*, *W*_*r*_^(*l*)^ is a layer-specific weight matrix with edge *r*, *W*_0_^(*l*)^ is the self-loop weight, and *σ* is an activation function. Both GCN and R-GCN can transform the dimensions of the embedding, so the output of the BERT encoder can be transformed to the required dimensions.

The initialized representations **e**_*d*_ and **e**_*ner*_ are the input of GCN. After the semantic propagation in the network, the output will contain the information of the original entity and the text entity itself, which also incorporates the information of the surrounding neighbor nodes.

In contrast to existing models that integrate text information, SP-TAG uses the pretraining model BERT to better express the semantic information of nodes in a text-augmented KG. With GCN-based semantic propagation, adjacent entities and text entities can share semantics.

In the following experiments, we will also compare the influence of whether the GCN is used for semantic propagation on the results. Details are provided in Section 5.3.

### 3.3. Joint Embedding

To preserve both the structural semantic information of the triples and the text semantic information of the entity descriptions, SP-TAG adopts a gate mechanism [32] to combine the two parts and obtain the final representation of the entity. When the two representations are combined, the weights in each dimension of the vector are automatically learned without manual parameter setting. Moreover, the gate vectors learned for different entities are different. In addition, the entire model can be trained in an end-to-end manner. For entity *e*, the expression for combining its structure- and text-based representations are as follows:

(5) $$\begin{array}{c} e=g_{e}⊙e_{s}+\left(1-g_{e}\right)⊙e_{d}\text{,} \end{array}$$

where **e**_*s*_ and **e**_*d*_ are the structure- and text-based representations of the entity, respectively, **g**_*e*_ is the gate that balances the two representations, and ⊙ is element-wise multiplication.

To constrain the value of each element in **g**_*e*_ to [0, 1], we use a sigmoid function as activation, i.e.,

(6) $$\begin{array}{c} g_{e}=\sigma \left({\tilde{g}}_{e}\right)\text{,} \end{array}$$

where ${\tilde{g}}_{e}$ is a vector specific to entity *e* and is simultaneously initialized and optimized with **e**_*s*_ and **e**_*d*_. The representations of the head entity, relationship, and tail entity are as follows:

(7) $$\begin{array}{c} h=g_{h}⊙h_{s}+\left(1-g_{h}\right)⊙h_{d}\text{,}\\ r=r_{d}\text{,}\\ t=g_{t}⊙t_{s}+\left(1-g_{t}\right)⊙t_{d}\text{.} \end{array}$$

In KGRL research, the construction of negative samples is an important aspect of training, and the quality of their construction affects model performance. For example, during training, many triples are easily judged to be wrong by the model, and sampling these triples does not provide new information for training. To obtain high-quality negative samples, Sun et al. [11] proposed a self-adversarial sampling method that performs dynamic sampling according to the representation of entities and relations, so that new negative samples contain new information. The method samples negative triples according to the probability distribution:

(8) $$\begin{array}{c} p\left(h_{j}^{\prime },r,\left.t_{j}^{\prime }\right|\left\{\left\{\left(h_{i},r_{i},t_{i}\right)\right\}\right.\right)=\frac{\exp\;\alpha f_{r}\left(h_{j}^{\prime },t_{j}^{\prime }\right)}{\sum_{i}\exp\;\alpha f_{r}\left(h_{i}^{\prime },t_{i}^{\prime }\right)}\text{,} \end{array}$$

where *α* is the sampling rate and *f*(*h*_*i*_′, *t*_*i*_′) is the score of the triple. This probability is also introduced into the loss function as the weights of the triples. The overall loss function of the model is as follows:

(9) $$\begin{array}{c} L=-\log\;\sigma \left(\gamma -f\left(h,t\right)\right)-\overset{n}{\underset{i=1}{\sum }}p\left(h_{i}^{\prime },r,t_{i}^{\prime }\right)\log\;\sigma \left(f\left(h_{i}^{\prime },t_{i}^{\prime }\right)-\gamma \right)\text{,} \end{array}$$

where *γ* is the margin hyperparameter, *σ* is the sigmoid function, and (*h*_*i*_′, *r*, *t*_*i*_′) is the *i*th negative sample. The model is trained to minimize the loss function, optimize parameters, and improve the quality of the entity and relation embedding.

## 4. Results and Discussion

We evaluated the model performance using a typical link prediction task. We also outperformed a hyperparameter analysis, ablation study, and semantic augmentation verification with a small number of training samples.

### 4.1. Experiment Setup

#### 4.1.1. Baselines

The comparative baseline models used in this experiment fall into two categories: triple-based methods (TransE [9], TransH [10], ConvE [12], ConvKB [25], R-GCN [40], RotatE [11], and MRotatE [24]) as well as methods that integrate text information (DKRL [14], Jointly [32], TEKE_E [43], AATE_E [35], ConMask [15], TA-ConvKB [36], BCRL [44], Pretrain-KGE [37], and TEGER [16]).

#### 4.1.2. Datasets

We used the datasets FB15K and WN18 [45], from the two real-world KGs Freebase and WordNet (Table 2). In addition to the triples, they include a specific text description of each entity. FB15K and WN18 have been widely used. However, several researchers argued that there are many inverse relations in FB15K and WN18 that cause data leakage. Therefore, these inverse relations were removed, yielding FB15K-237 [46] and WN18RR [12].

#### 4.1.3. Evaluation Metrics

The evaluation metrics mean rank (MR), mean reciprocal rank (MRR), and HITS@N were used. MR reflects the average ranking of correct triples in all prediction results, where smaller values indicate better performance, MRR reflects the average of the reciprocal rankings of all correct triples, where larger values indicate better performance, and HITS@N reflects the correct triples in the top *n* results, where larger values indicate better performance.

#### 4.1.4. Implementations

Depending on the dataset, the ranges of some parameter values vary. For FB15K and FB15K-237, vector dimension is defined as *d* ∈ {600,700,800} and margin hyperparameter is defined as *γ* ∈ {6,12,18}. For WN18RR and WN18, vector dimension is defined as *d* ∈ {80,100,200} and margin hyperparameter is defined as *γ* ∈ {6,9,12}. In the selection of the remaining parameters, sampling rate is defined as *α* ∈ {0.5,1.0} and learning rate is defined as *λ*=0.00005. When using BERT to encode text, the maximum truncation length for text entity descriptions was set to 100 words. We use Adam [47] to optimize the parameters.

### 4.2. Results and Analysis

#### 4.2.1. Link Prediction

We evaluated the performance of the method by predicting the missing head or tail entities in triples. We tested the performance of SP-TAG with two representative methods TransE and RotatE and compared it with the baselines introduced above, making the results more convincing and trustworthy. Tables 3 and 4 list the results of SP-TAG and the baselines. Methods above the horizontal solid line only consider triples, whereas those below the line integrate text information.

The results in Tables 3 and 4 yield the following observations:

1. Compared with the triple-based methods, methods integrating text information generally perform better with respect to MR and HITS@10, whereas for HITS@1, HITS@3, and MRR, they also perform competitively. This reveals that text information effectively improves the lower bound of the entity link prediction ranking and improves average prediction performance.
2. Compared with the original models TransE and RotatE, the SP-TAG-based models perform significantly better, demonstrating that text information brings more semantic features to entities. When the triple structure information is insufficient, text is a powerful complement that improves the performance of representation learning.
3. Though TransE is an early method, SP-TAG-TransE can still achieve excellent results and is comparable with recent models, demonstrating that classical models can achieve very good results through KG augmentation and semantic propagation. Taking WN18RR as an example, the MR of the SP-TAG-TransE model (1423) is still substantially better than those of recent models (>2000), and the difference with respect to SP-TAG-RotatE is quite small. These models obtain the top two results.
4. SP-TAG-RotatE achieved the best MR, HITS@3 and HITS@10 results of all methods on FB15K-237, WN18, and WN18RR, and its MRR, HITS@1 were among the top results. On FB15K-237, the improvement obtained by SP-TAG is not as obvious as it is on WN18RR. However, considering the performance of the original method, the improvement is substantial, reaching an average level. We believe that because of the more complex scale and structure of FB15K-237, the triple structure contains more information than WN18RR itself, so the augmentation and semantic propagation effects are not as obvious.
5. Because FB15K-237 and WN18RR have no inverse relations, link prediction on these datasets is more difficult. On these datasets, SP-TAG's performance metrics decrease less and are more stable than those of Pretrain-KGE. The MR of Pretrain-RotatE decreased from 125 on WN18 to 2138 on WN18RR, whereas the MR of SP-TAG-RotatE only decreased from 72 to 942. On FB15K and FB15K-237, they performed comparably. Hence, when the complexity of the dataset increases, SP-TAG has better stability and adaptability because of the augmentation of the KG and semantic propagation.

#### 4.2.2. Parameter Analysis

**Figure 5:**
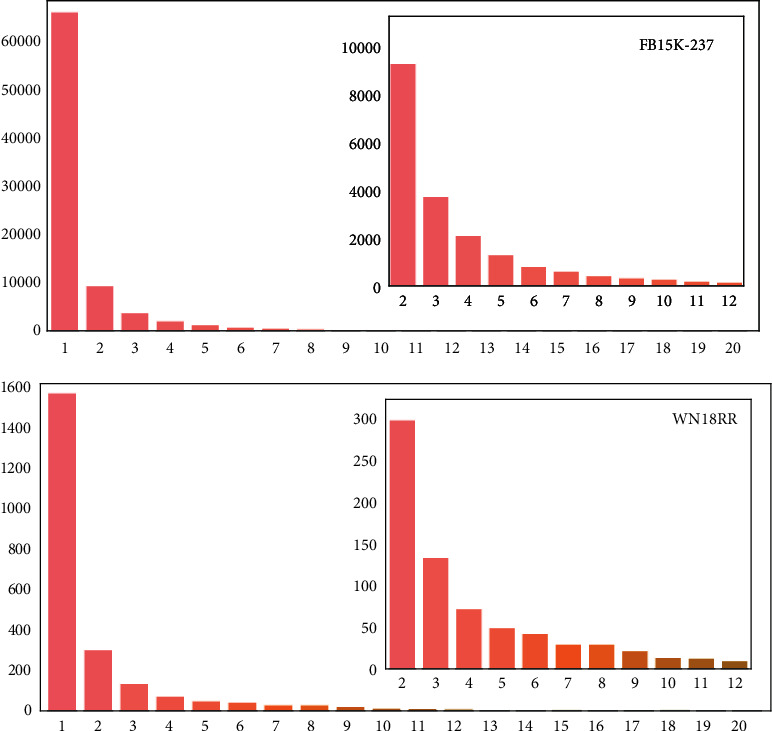
Distribution of the number of edges between the text entities and original entities in *K*_TAG_.

To analyse the impact of semantic propagation on *K*_TAG_, SP-TAG-RotatE is used to analyse hyperparameter *k*.  Figure 5 shows the distribution of the numbers of original entities connected to each text entity in *K*_TAG_. The abscissa is the number of connected entities, and the ordinate is the number of corresponding text entities.

In each *K*_TAG_, many text entities connect to only one entity. More than 1,500 such text entities exist in WN18RR (14.9% of all text entities) and more than 60,000 exist in FB15K-237 (30% of all text entities). As mentioned in previous section, retaining such text entities does not propagate semantics between entities and increases overall complexity. The small histograms on the right show that the number of text entities is roughly inversely proportional to the number of connected entities.

**Figure 6:**
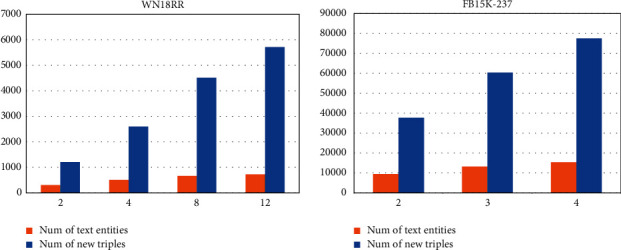
Number of entities and triples added to *K*_TAG_ under different parameters *k*.

The descriptions of entities in FB15K-237 are longer and more detailed than in WN18RR, so more text entities can be extracted to enhance the KG, and we tend to use a smaller *k* for FB15K-237 and a larger *k* for WN18RR. When choosing the value of *k*, we set *k* to {2,3,4} for FB15K-237 and {2,4,8,12} for WN18RR.  Figure 6 lists the specific number of text entities and the number of new triples in *K*_TAG_ under different *k*, where the abscissa is the hyperparameter *k*, and the two histograms represent the number of new text entities and the number of new triples.

Table 5 further compares the numbers of nodes, edges, and average edges per node of *K* and the corresponding *K*_TAG_ for different values of *k*. After the augmentation, the number of edges (i.e., number of triples) has been increased to a certain extent. For example, the average number of edges per node in WN18RR increased by 6.6%, and that in FB15K-237 increased by 28.5%. Therefore, the entities are associated more tightly, and semantics are fully propagated on *K*_TAG_.

**Figure 7:**
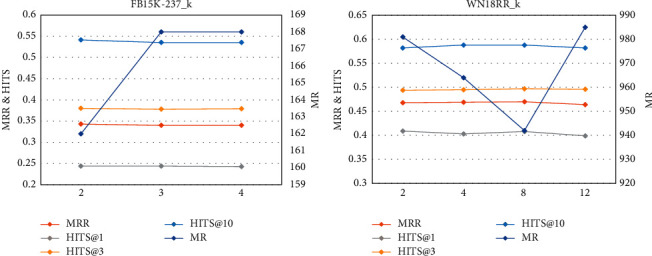
Line charts of parameter *k* analysis on FB15K-237 and WN18RR.

Figure 7 and Table 6 show the results of link prediction for FB15K-237 and WN18RR when *k* varies. Of the five metrics, MR is the most sensitive to hyperparameter *k*. On FB15K-237, as *k* increases, the link prediction metrics slightly decrease. On WN18RR, as *k* gradually increases from 2 to 8, the link prediction performance improves, but when *k* further increases to 12, the results are worse. This is due to the overpropagation of semantics. The addition of too many text entities induces noise, as described before. Hence, it is important to choose the number of text nodes when constructing *K*_TAG_.

**Figure 8:**
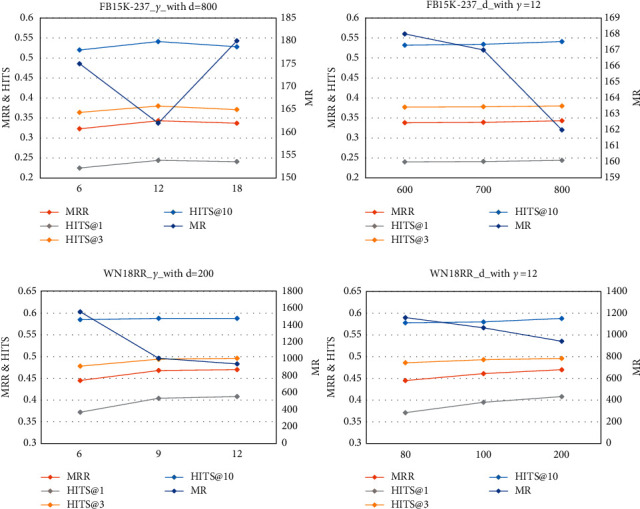
Line charts of parameter *d* and *γ* analysis on FB15K-237 and WN18RR.

The remaining main hyperparameters are dimension *d* and margin *γ*. For SP-TAG-RotatE, we used a grid search to select the optimal parameters (FB15K-237: *γ*=12, *d*=800 and WN18RR: *γ*=12, *d*=200).  Figure 8 and Tables 7 and 8 present the results when one of the parameters is fixed and the other is adjusted (the remaining unrelated parameters are fixed).

The results reveal that

1. For both datasets, the effect of *γ* on the results is obvious. When *γ* is optimized, the performance of the model significantly improves (WN18RR, *γ*=12). However, if the value is not appropriate, the performance decreases (FB15K-237, *γ*=18).
2. On both datasets, as the dimension increases, the performance of the model improves, indicating that vectors with higher dimensions are important for fully expressing the features of entities and relations. The dimension parameter is typically related to dataset complexity. In terms of scale and content, FB15K-237 is more complex than WN18RR, and hence to represent the features of entities and relations in this dataset, a higher dimension is required.
3. The effect of the parameter values is more pronounced on WN18RR. For simple datasets, finding appropriate parameters can effectively improve the performance of the model, whereas for more complex datasets, additional factors must be considered.

To explore the influence of hyperparameters on datasets from the same KG with different distributions, we also used SP-TAG-RotatE to compare the results of WN18 and WN18RR for different values of *d* and *γ*.

**Figure 9:**
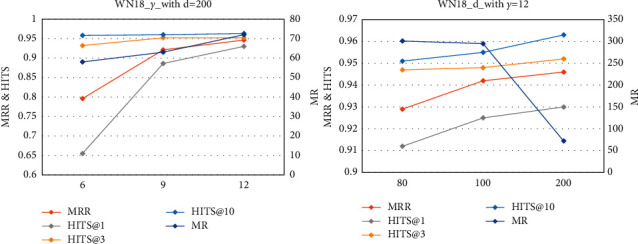
Line charts of parameter *d* and *γ* analysis on WN18.

Figure 9 reveals that as the dimension increases, MR is significantly improved, whereas the other four metrics slightly improve. With the increase in *γ*, MRR and HITS@1 significantly improve, and HITS@3 and HITS@10 slightly improve.

The results of MRR and HITS@1 in Table 9 show that *γ* still has a significant impact on this dataset. Unlike on WN18RR, on WN18, *d* is an important factor. In addition, parameter adjustment has a greater impact on WN18 (MRR increased by 18.8% from 0.796 to 0.946, and MR decreased by 76.1% from 301 to 72), whereas WN18RR was relatively less sensitive (MRR increased by 5.6% from 0.445 to 0.47, and MR decreased by 18.8% from 1160 to 942). As mentioned above, a large number of inverse relations in WN18 were removed to create WN18RR, making the WN18RR dataset more complex; in other words, WN18 has more data containing more information, making WN18 simpler. Consistent with previous experimental results, the simpler the dataset, the greater the impact of parameter tuning on the results. The data distributions are different, and thus the hyperparameters have different effects on them.

#### 4.2.3. Ablation Study

We evaluated the effect of GCN and R-GCN in semantic propagation as well as the importance of semantic propagation by replacing GCN with a linear transform. The linear transform is obtained by a matrix operation *M*_*p∗q*_, where *p* represents the embedding dimension and *q* represents the output dimension of the BERT encoder. Finally, we demonstrate the importance of textual information and the gate mechanism by replacing the BERT text initialization vector with a random vector and the gate vector with a constant.

Table 10 reveals that on both FB15K-237 and WN18RR, when the dimensions are the same, the effect of using GCN for semantic propagation is not as good as that of R-GCN, indicating that for each relation, setting a different weight matrix to distinguish their semantics improves results. Moreover, when R-GCN is used instead of the linear transform, the model has significant advantages in MRR, HITS@1, and HITS@3, demonstrating that semantic propagation further improves the original top-ranked prediction results.

Table 11 reveals that on both datasets, the gate mechanism helps balance the text- and structure-based representations, and the semantic information introduced by BERT effectively improves the representation learning performance of entities.

#### 4.2.4. Verifying Semantic Augmentation with Few Training Samples

To further illustrate how SP-TAG more fully utilizes the semantic information in text, we reduced the number of training samples in the WN18RR dataset. The total number of entities remained the same, but the number of triples was reduced by 60%.

To make the comparison more convincing, the DKRL method (BERT + TransE), which also integrates textual information, was evaluated to highlight the importance of the text-augmented KG and semantic propagation when the number of training samples is limited.

The results in Table 12, demonstrate that the models DKRL and SP-TAG combined with text information perform significantly better than TransE, which only uses triples in link prediction. When there are fewer training samples, the models obtain more entity feature information from the text, effectively compensating for the lack of triple training samples. Compared with the performance of DKRL, the performance of SP-TAG is obviously better in all three metrics. Its MR is very close to the results obtained by some methods on the complete training set (e.g., Pretrain-RotatE). These results further demonstrate that SP-TAG more closely connects entities in KG by connecting text entities and better achieves semantic propagation between related entities.

## 5. Conclusion and Future Work

To address the problem of insufficient utilization of text semantic information in existing methods, we proposed SP-TAG, which is based on text-augmented KG semantic propagation to better realize the full integration of text semantics and structural semantics and further improve the utilization of text information. The experimental analysis on multiple benchmark datasets demonstrated that SP-TAG can effectively improve link prediction performance, especially when the number of training samples is limited.

Our experimental results demonstrate the feasibility of the theory and indicate the significance of continuing research in this direction. In the future, the following further improvements could be considered:

1. When building a text-augmented KG, the entities and text entities could be aligned to make the KG more streamlined and accurate.
2. During semantic propagation, the attention mechanism can be further combined to obtain different representations of entities for different relations.
3. For entities that do not appear in the training set, text information could be used to represent them to achieve zero-shot prediction or open KG prediction.

## Figures and Tables

**Table 1:** Extracted named entity types.

Type	Abbr	Description
1	Person	People
2	NORP	Nationality, religion, etc.
3	FAC	Building, airport, etc.
4	ORG	Company, agency, etc.
5	GPE	Country, city, etc.
6	LOC	Mountain, water, etc.
7	Product	Objects, vehicles, etc.
8	Event	Battle, war, event, etc.
9	Work_of_art	Book, song, etc.
10	Law	Document made into law
11	Language	Named language

**Table 2:** Statistics of the datasets.

Dataset	# entities	# relations	# train	# valid	# test
FB15K	14,951	1,344	483,142	50,000	59,071
FB15K-237	14,541	237	272,115	17,535	20,466
WN18	40,943	18	141,442	5,000	5,000
WN18RR	40,943	11	86,835	3,034	3,134

**Table 3:** Results on FB15K and WN18.

Method	FB15K					WN18				
	MR	MRR	HITS @1	HITS @3	HITS @10	MR	MRR	HITS @1	HITS @3	HITS @10
TransE **[†]**	—	0.463	0.297	0.578	0.749	—	0.495	0.113	0.888	0.943
TransH **[†]**	40	0.734	0.651	0.796	0.867	284	0.719	0.598	0.806	0.923
ConvE **[†]**	51	0.657	0.558	0.723	0.831	374	0.943	0.935	0.946	0.956
R-GCN	—	0.651	0.541	0.736	0.825	—	0.814	0.686	0.928	0.955
RotatE **[†]**	40	*0.797*	*0.746*	*0.830*	0.884	309	*0.949*	*0.944*	**0.952**	0.959
MRotatE **[†]**	46	**0.807**	**0.762**	**0.833**	*0.886*	272	**0.950**	**0.945**	**0.952**	0.959
DKRL	113	—	—	—	0.576	—	—	—	—	—

Jointly (A-LSTM)	77	—	—	—	0.755	123	—	—	—	0.909
TEKE_E	79	—	—	—	0.676	127	—	—	—	0.938
AATE_E	76	—	—	—	0.761	123	—	—	—	—
ConMask (head)	116	—	—	—	0.620	—	—	—	—	—
ConMask (tail)	80	—	—	—	0.620	—	—	—	—	—
BCRL	67	—	—	—	0.823	90	—	—	—	0.949
Pretrain-TransE	**37**	0.731	—	—	0.866	*85*	0.757	—	—	0.928
Pretrain-RotatE	*38*	0.784	—	—	0.881	125	0.927	—	—	0.962
TEGER-TransE	72	—	—	—	0.763	168	—	—	—	0.947
TEGER-ConvE	47	—	—	—	0.851	336	—	—	—	0.956
SP-TAG-TransE	52	0.646	0.530	0.734	0.829	103	0.783	0.693	0.854	0.947
SP-TAG-RotatE	41	0.756	0.668	0.821	**0.888**	**72**	0.946	0.930	**0.952**	**0.963**

Best results are in bold and the second best results are italics. Results of [†] are taken from reference [24]. Other results come from the corresponding original papers.

**Table 4:** Results on FB15K-237 and WN18RR.

Method	FB15K-237					WN18RR				
	MR	MRR	HITS @1	HITS @3	HITS @10	MR	MRR	HITS @1	HITS @3	HITS @10
TransE **[†]**	347	0.294	—	—	0.465	3384	0.226	—	—	0.501
TransH **[†]**	173	0.331	0.232	0.371	0.529	3748	0.212	0.008	0.386	0.496
ConvE **[†]**	244	0.325	0.237	0.356	0.501	4187	0.430	0.390	0.430	0.520
ConvKB **[††]**	257	*0.406*	—	—	0.517	1754	0.248	—	—	0.520
RotatE **[†]**	177	0.338	*0.241*	*0.375*	0.533	3340	*0.476*	*0.428*	*0.492*	0.571
MRotatE **[†]**	195	0.333	0.238	0.368	0.524	4890	**0.477**	**0.440**	0.488	0.552

TA-ConvKB **[††]**	248	**0.426**	—	—	*0.539*	1360	0.267	—	—	0.568
Pretrain-TransE	**162**	0.332	—	—	0.529	1747	0.235	—	—	0.557
Pretrain-RotatE	*168*	0.337	—	—	0.534	2138	0.447	—	—	*0.580*
SP-TAG-TransE	171	0.330	0.232	0.375	0.530	*1423*	0.239	0.210	0.401	0.537
SP-TAG-RotatE	**162**	0.343	**0.244**	**0.380**	**0.541**	**942**	0.470	0.408	**0.497**	**0.588**

Best results are in bold and the second best results are italics. Results of [†] are taken from reference [24] and results of [††] are taken from reference [36]. Other results come from the corresponding original papers.

**Table 5:** Statistics of original *KG* and *K*_TAG_.

Dataset	*k*	# nodes		# edges		Average edges per node		
		Before	After	Before	After	Before	After	Lift (%)
FB15K-237	2	14,541	+9424	272,115	+37696	18.71	21.31	13.9
	3		+13212		+60424		22.87	22.2
	4		+15347		+77504		24.04	28.5

WN18RR	2	40,943	+301	86,835	+1204	2.12	2.15	1.4
	4		+509		+2598		2.18	3.0
	8		+662		+4514		2.23	5.2
	12		+721		+5716		2.26	6.6

**Table 6:** Results of parameter*k* analysis on FB15K-237 and WN18RR.

*k*	FB15K-237			WNRR18			
	*k*=2	*k*=3	*k*=4	*k*=2	*k*=4	*k*=8	*k*=12
MRR	**0.343**	0.340	0.340	0.468	0.469	**0.470**	0.464
HITS@1	**0.244**	0.244	0.243	**0.409**	0.403	0.408	0.399
HITS@3	**0.380**	0.378	0.379	0.494	0.495	**0.497**	0.496
HITS@10	**0.541**	0.535	0.535	0.582	0.588	**0.588**	0.582
MR	**162**	168	168	981	964	**942**	985

Best results are in bold.

**Table 7:** Results of parameter *γ* analysis on FB15K-237 and WN18RR.

*γ*	FB15K-237			WNRR18		
	*γ*=6	*γ*=12	*γ*=18	*γ*=6	*γ*=9	*γ*=12
MRR	0.323	**0.343**	0.337	0.448	0.468	**0.470**
HITS@1	0.225	**0.244**	0.241	0.372	0.404	**0.408**
HITS@3	0.364	**0.380**	0.371	0.478	0.494	**0.496**
HITS@10	0.520	**0.541**	0.528	0.585	0.588	**0.588**
MR	175	**162**	180	1559	1009	**942**

Best results are in bold.

**Table 8:** Results of parameter *d* analysis on FB15K-237 and WN18RR.

*d*	FB15K-237			WNRR18		
	*d*=600	*d*=700	*d*=800	*d*=80	*d*=100	*d*=200
MRR	0.338	0.339	**0.343**	0445	0.461	**0.470**
HITS@1	0.240	0.241	**0.244**	0.371	0.395	**0.408**
HITS@3	0.377	0.378	**0.380**	0.486	0.493	**0.496**
HITS@10	0.532	0.534	**0.541**	0.578	0.580	**0.588**
MR	168	167	**162**	1160	1066	**942**

Best results are in bold.

**Table 9:** Results of parameter *d* and *γ* analysis on WN18.

	FB15K-237			WNRR18		
	*γ*=6	*γ*=9	*γ*=12	*d*=80	*d*=100	*d*=200
MRR	0.796	0.921	**0.946**	0.929	0.942	**0.946**
HITS@1	0.655	0.886	**0.930**	0.912	0.925	**0.930**
HITS@3	0.932	0.952	**0.952**	0.947	0.948	**0.952**
HITS@10	0.958	0.960	**0.963**	0.951	0.955	**0.963**
MR	**58**	63	72	301	295	**72**

Best results are in bold.

**Table 10:** Effect of semantic propagation on performance.

Method	FB15K-237 SP-TAG-RotatE				
	MR	MRR	HITS@1	HITS@3	HITS@10

w/R-GCN	172	0.322	0.226	0.361	0.515
w/GCN	185	0.310	0.220	0.352	0.508
w/GCN [†]	162	**0.343**	**0.244**	**0.380**	**0.541**
w/linear [†]	**153**	0.337	0.240	0.373	0.534

Method	WN18RR SP-TAG-RotatE				
	MR	MRR	HITS@1	HITS@3	HITS@10

w/R-GCN	942	**0.470**	**0.408**	**0.497**	0.588
w/GCN	2080	0.197	0.015	0.319	0.549
w/linear	**802**	0.453	0.376	0.493	**0.595**

[†] denotes that the embedding dimension is 800, whereas the others are 200. Best results are in bold.

**Table 11:** Effect of the gate mechanism and BERT encoder on performance.

Method	FB15K-237 SP-TAG-RotatE				
	MR	MRR	HITS@1	HITS@3	HITS@10

Baseline	**172**	**0.322**	**0.226**	**0.361**	**0.515**
w/o GATE	196	0.316	0.221	0.351	0.507
w/o BERT	261	0.300	0.212	0.330	0.476

Method	WN18RR SP-TAG-RotatE				
	MR	MRR	HITS@1	HITS@3	HITS@10

Baseline	**942**	**0.470**	0.408	**0.497**	**0.588**
w/o GATE	2551	0.450	0.392	0.477	0.561
w/o BERT	4822	0.456	**0.421**	0.468	0.524

Best results are in bold.

**Table 12:** Link prediction results with few training samples.

Method	MR	MRR	HITS@10
TransE	9738	0.138	0.339
DKRL (BERT + TransE)	4343	0.139	0.349
SP-TAG-TransE	**2248**	**0.145**	**0.371**

Best results are in bold.

## Data Availability

The data used to support the findings of this study are available from the corresponding author upon request.
